# Changes in weight loss, body composition and cardiovascular disease risk after altering macronutrient distributions during a regular exercise program in obese women

**DOI:** 10.1186/1475-2891-9-59

**Published:** 2010-11-22

**Authors:** Chad M Kerksick, Jennifer Wismann-Bunn, Donovan Fogt, Ashli R Thomas, Lem Taylor, Bill I Campbell, Colin D Wilborn, Travis Harvey, Mike D Roberts, Paul La Bounty, Melyn Galbreath, Brandon Marcello, Christopher J Rasmussen, Richard B Kreider

**Affiliations:** 1 Applied Biochemistry and Molecular Physiology Laboratory, Health and Exercise Science Department, University of Oklahoma, Norman, OK 73019-6081, USA; 2 Endocrinology and Diabetes Section, Department of Pediatrics, University of Oklahoma Health Sciences Center, Oklahoma City, OK 73104, USA; 3 Department of Exercise Science, Campbell University, Buies Creek, NC 27502, USA; 4 Laboratory for Exercise Biochemistry and Metabolism Health and Kinesiology Department, College of Education and Human Development, San Antonio, TX 78249, USA; 5 Department of Health, Human Performance and Recreation, Baylor University, Waco, TX 76798-7313, USA; 6 Human Performance Lab, Exercise & Sport Science Department, University of Mary-Hardin Baylor, Belton, TX 76513, USA; 7 School of Physical Education & Exercise Science, University of South Florida, Tampa, FL 33620, USA; 8 75th Ranger Regiment, Ranger Athlete Warrior, 6420 Dawson Loop, Fort Benning, GA 31905-4625, USA; 9 Dept of Biomedical Sciences, University of Missouri-Columbia, 1600 E. Rollins, Columbia, MO 65211, USA; 10 King Faisal Heart Institute, MBC 16, KFSH&RC, P.O. Box 3354, Riyadh, 11211, Saudi Arabia; 11 Department of Athletics, Stanford University, Palo Alto, CA 94305, USA; 12 Department of Health & Kinesiology, Texas A & M University, College Station, TX 77843, USA

## Abstract

**Background:**

This study's purpose investigated the impact of different macronutrient distributions and varying caloric intakes along with regular exercise for metabolic and physiological changes related to weight loss.

**Methods:**

One hundred forty-one sedentary, obese women (38.7 ± 8.0 yrs, 163.3 ± 6.9 cm, 93.2 ± 16.5 kg, 35.0 ± 6.2 kg•m^-2^, 44.8 ± 4.2% fat) were randomized to either no diet + no exercise control group (CON) a no diet + exercise control (ND), or one of four diet + exercise groups (high-energy diet [HED], very low carbohydrate, high protein diet [VLCHP], low carbohydrate, moderate protein diet [LCMP] and high carbohydrate, low protein [HCLP]) in addition to beginning a 3x•week^-1 ^supervised resistance training program. After 0, 1, 10 and 14 weeks, all participants completed testing sessions which included anthropometric, body composition, energy expenditure, fasting blood samples, aerobic and muscular fitness assessments. Data were analyzed using repeated measures ANOVA with an alpha of 0.05 with LSD post-hoc analysis when appropriate.

**Results:**

All dieting groups exhibited adequate compliance to their prescribed diet regimen as energy and macronutrient amounts and distributions were close to prescribed amounts. Those groups that followed a diet and exercise program reported significantly greater anthropometric (waist circumference and body mass) and body composition via DXA (fat mass and % fat) changes. Caloric restriction initially reduced energy expenditure, but successfully returned to baseline values after 10 weeks of dieting and exercising. Significant fitness improvements (aerobic capacity and maximal strength) occurred in all exercising groups. No significant changes occurred in lipid panel constituents, but serum insulin and HOMA-IR values decreased in the VLCHP group. Significant reductions in serum leptin occurred in all caloric restriction + exercise groups after 14 weeks, which were unchanged in other non-diet/non-exercise groups.

**Conclusions:**

Overall and over the entire test period, all diet groups which restricted their caloric intake and exercised experienced similar responses to each other. Regular exercise and modest caloric restriction successfully promoted anthropometric and body composition improvements along with various markers of muscular fitness. Significant increases in relative energy expenditure and reductions in circulating leptin were found in response to all exercise and diet groups. Macronutrient distribution may impact circulating levels of insulin and overall ability to improve strength levels in obese women who follow regular exercise.

## Introduction

The prevalence of obesity in the U.S and throughout the world continues to increase. Comprehensive reviews on the topic estimate that 1.2 billion people in the world are overweight and at least 300 million of them are obese [1]. While largely thought to be preventable, obesity is linked to an estimated 300,000 deaths in the U.S. every year. Not just concerns related to excessive weight, obesity is also strongly linked to other disorders such as hypertension, diabetes, hypercholesterolemia, and liver disease. As a result, much research is being conducted worldwide to help identify causative mechanisms as well as programs to better manage its progression. Recently, many investigations have focused on increasing the proportion of dietary protein relative to carbohydrate to examine changes in weight loss, body composition and energy expenditure as well as various serum markers of substrate utilization and appetite regulation [2-5]. Many of these studies have reported these types of diets to have positive effects on weight loss as well as markers of disease risk, which include body composition, blood lipids and glucose and insulin kinetics [2,6,7], while other studies in obese populations have reported no difference in weight loss [8], but favorable effects on other markers of disease risk [5,9,10].

A higher protein intake in overweight and obese populations has been indicated for many reasons, including a better regulation of glucose and insulin homeostasis and prevention of lean muscle loss; an effect closely related with programs of substantial reductions in dietary energy intake [6,7]. Studies which incorporated insulin resistant, metabolic syndrome or those diagnosed as with type II diabetes reported that diets with a higher protein intake stimulate greater weight loss and improvements in glucose homeostasis and cholesterol status [2,5] as well as overall improvements in the incidence of metabolic syndrome [4,11]. Additionally, much interest in various adipokines (e.g. resistin, leptin, adiponectin) has been generated due to their relationship to appetite, energy expenditure, insulin sensitivity and cardiovascular disease [12,13]. While many studies have reported upon the relationship between acute changes in circulating levels of leptin in healthy [13], overweight [3,14,15] and diabetic populations [16], the impact of these responses relative to higher protein and higher carbohydrate requires additional investigation.

In addition to dietary modifications, the inclusion of more physical activity is often indicated to stimulate weight loss [17-21], increase energy expenditure [19] and promote improvements in insulin sensitivity [20,21] as well as other indicators of cardiovascular disease risk [17,19-21]. While much research has been conducted highlighting the benefits of even modest amounts of physical activity, a small number of studies have reported upon the impact of an exercise program with various dietary interventions of which can include caloric restriction and alterations in the ratio of dietary protein and dietary carbohydrate [3,4,22,23]. Furthermore, even less scientific literature has reported upon physiological as well as biochemical adaptations that occur following this type of dieting in combination with resistance-based exercise [3,22]. While cardiovascular forms of exercise are more popular, findings from these studies suggest that combining dietary interventions with a resistance-based exercise program promotes changes in weight loss and improvements in risks for cardiovascular disease, but also attenuate losses in lean mass which commonly occurs in dietary programs which severely restrict energy intake [19,22] as well as promote a greater maintenance of energy expenditure [3,19] and insulin sensitivity [24]. The present study is the second of a series of investigations conducted by our research group to examine the changes in body composition, fitness and health while participating in a weekly, resistive exercise program. Two major advances were studied in the present investigation. A shorter hypocaloric period (one week vs. two weeks) was utilized as a means to stimulate weight loss but minimize the negative metabolic influence seen in the first investigation [3]. Second, a modified weight maintenance approach was adopted over the last four weeks of the present study. The ideal outcome from these modifications were to develop a diet and exercise program that promoted a healthy amount of weight loss that could be sustained without negative and otherwise untoward effects of the program itself (e.g. maintenance of fat-free mass and energy expenditure) [25].

For these reasons, the purpose of this study was to elucidate the impact of different macronutrient distributions in conjunction with a regular exercise program and to further examine dietary strategies which complement the workout regimen employed by this investigation. The specific aims of this study were to determine the impact of combining various dietary interventions with a resistance exercise program on changes in weight loss, body composition, cardiovascular and muscular fitness parameters, resting energy expenditure, and serum markers of clinical safety and substrate utilization in sedentary, obese women. It was hypothesized that all exercise and diet groups would significantly lose weight and experience significant improvements in their fitness. Further, we hypothesized that consumption of a diet with a higher proportion of dietary protein would further stimulate positive adaptations in body composition and markers of cardiovascular disease risk [22].

## Methods

### Experimental Approach

This study was designed as a follow-up to an initial study by our group [3] which was designed to assess the overall safety and efficacy of following the exercise and diet recommendations of the Curves program. The present follow-up study sought to determine the impact of an initial one week bout of intensive caloric restriction of varying macronutrient ratios followed by a moderate caloric restriction diet of varying macronutrient ratios while participating in a regular exercise program. In concert with our previous research design, participants were matched into clusters according to age and body mass and placed into one of six diet and exercise combinations [3].

**Figure 1:**
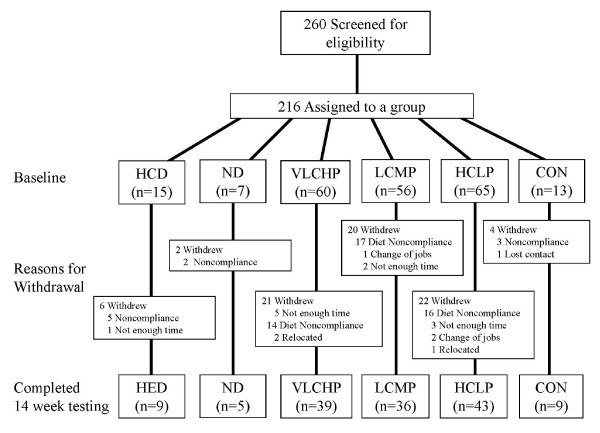
**Standard consort diagram**. Highlighted divisions include those people who responded to announcement, those who were screened, group assignments, those who completed program and reasons for termination.

The first phase of dieting (Phase I) lasted one week. During this time, participants were assigned to follow the prescribed exercise program and follow one of four diet + exercise combinations (presented as kcals; % carbohydrate: protein: fat): 1) a high energy, high carbohydrate, low protein diet (HED) [2,600; 55:15:30%], 2) a very low carbohydrate, high protein diet (VLCHP) [1,200; 7:63:30%], 3) a low carbohydrate, moderate protein diet (LCMP) [1,200; 20:50:30%], or 4) a high carbohydrate, low protein diet (HCLP) [1,200; 55:15:30%]. Phase II lasted for last for nine weeks, increased caloric intake in all restricted groups to 1,600 calories per day and changed the macronutrient ratios of VLCHP and HCLP to 1,600; 15:55:30%. Further, the HED group altered their macronutrient ratio to 2,600; 40:30:30%. Phase III was a four week period which had all participants follow the same diet (2,600; 55:15:30%) and participants were instructed to weigh themselves each day. Upon gaining three pounds, participants were told to follow their phase I diet until the additional weight was lost. All subjects were tested over the course of a 14 week period at 0, 1, 10, and 14 weeks to determine any changes in criterion variables. We hypothesized throughout this study that participating in the diet and exercise programs would facilitate weight loss in addition to improvements in various indicators of health in comparison to the control groups (CON and ND+E). Two additional aims were present which included a continued investigation into the impact caused by alterations in the macronutrient ratio (higher protein vs. higher carbohydrate) for their efficacy at facilitating weight loss and promoting health. In this regard, we have hypothesized that those dietary groups which contained higher amounts of dietary protein would experience smaller reductions in lean tissue mass in comparison to other diet groups. The remaining aim was centered on acute alterations in caloric intake and its impact on energy expenditure, selected metabolic hormones and subsequent weight loss and health changes. Towards this aim we hypothesized that acute alterations would facilitate weight maintenance while allowing for periods of higher and lower caloric intake. According to these hypotheses, primary outcomes in this study were identified as waist circumference; secondary outcomes were body mass and DXA parameters while tertiary outcomes were the changes seen in cardio respiratory and muscular fitness, resting energy expenditure, serum and whole blood safety and hormonal markers. A standard consort diagram is available which outlines the numbers of participants who were screened (n = 260), assigned to a group (n = 216), including how many to each intervention group and those that completed the entire 14 week protocol (n = 141) (see Figure 1).

### Subjects

One-hundred forty-one sedentary, obese women (38.7 ± 8.0 yrs, 163 ± 7 cm, 93.2 ± 16.5 kg, 35.0 ± 6.2 kg•m^-2^, 44.8 ± 4.2% fat) participated in this study. No baseline differences were exhibited amongst groups for primary, secondary and tertiary variables of interest, with the exception of resting energy expenditure (Table 1). Participants were not allowed to participate in this study if they reported at baseline any of the following situations: 1.) presence or diagnosis of any metabolic or cardiovascular disorder including known electrolyte abnormalities; heart disease, arrhythmias, diabetes, thyroid disease, or hypogonadism; a history of hypertension, hepatorenal, musculoskeletal, autoimmune, or neurologic disease; 2.) currently taking or prescribed medications for hyperlipidemia, hypoglycemia, hypertension, or androgenic medications; 3.) if they have taken ergogenic levels of nutritional supplements that may affect muscle mass (e.g., creatine, HMB), anabolic/catabolic hormone levels (androstenedione, DHEA, etc.), or weight loss (e.g., ephedra, thermogenics, etc.) within six months prior to the start of the study; 4.) were classified as high risk for cardiovascular disease according to American College of Sports Medicine criteria; 5) they agreed to not participate in any other form of a diet or exercise program during their participation in the study. All eligible participants signed informed consent statements approved by the institutional review board (IRB) for the protection of human subjects at Baylor University and were in compliance with the Declaration of Helsinki.

**Table 1:** Baseline anthropometric, body composition, biochemical parameters and dietary analysis for the high energy, high carbohydrate diet + exercise (HED; 2,600: 55:15:30), no diet + exercise (ND), very low carbohydrate, high protein diet + exercise (VLCHP: 1,200; 63:7:30), low carbohydrate, moderate protein diet + exercise (LCMP: 1,200; 50:20:30), high carbohydrate, low protein diet + exercise (HCLP: 1,200; 55:15:30) and no diet + no exercise control (CON).

*Demographics*	Grand Mean (n = 141)	HED (n = 9)	ND (n = 5)	VLCHP (n = 39)	LCMP (n = 36)	HCLP (n = 43)	CON (n = 9)	*P*-value
Age (years)	39 ± 8	43 ± 7	42 ± 2	38 ± 8	40 ± 7	38 ± 8	32 ± 10	0.06
Height (cm)	163 ± 7	162 ± 8	165 ± 3	163 ± 8	164 ± 7	164 ± 6	163 ± 6	0.95
Weight (kg)	93 ± 17	88 ± 16	93 ± 28	95 ± 18	95 ± 15	93 ± 15	86 ± 17	0.65
Body mass index (kg•m^-2^)	35 ± 6	33 ± 6	34 ± 11	36 ± 7	36 ± 6	35 ± 5	33 ± 6	0.70
Waist (cm)	98 ± 13	97 ± 6	86 ± 11	99 ± 15	102 ± 14	98 ± 11	92 ± 13	0.12
DXA fat-free mass (kg)	47 ± 7	46 ± 8	47 ± 11	48 ± 7	47 ± 6	48 ± 6	45 ± 7	0.87
DXA fat mass (kg)	39 ± 10	35 ± 9	41 ± 16	39 ± 10	42 ± 10	39 ± 9	35 ± 9	0.28
REE (kcals•d^-1^)	1632 ± 277	1297 ± 319	1462 ± 246	1690 ± 283^f^	1642 ± 249^f^	1642 ± 247^f^	1720 ± 265^f^	< 0.005
Systolic blood pressure (mm Hg)	123 ± 13	122 ± 13	118 ± 4	125 ± 13	122 ± 14	123 ± 13	121 ± 13	0.89
Diastolic blood pressure (mm Hg)	82 ± 8	82 ± 11	81 ± 8	84 ± 10	82 ± 8	82 ± 7	80 ± 7	0.84
VO_2 _(ml/kg/min)	22 ± 4	24 ± 3	21 ± 4	22 ± 4	21 ± 5	22 ± 3	24 ± 5	0.51
								
*Biochemical parameters*	Grand Mean	HED	ND	VLCHP	LCMP	HCLP	CON	*P*-value

Total cholesterol (mmol•L^-1^)	5.0 ± 0.9	5.2 ± 1.0	5.4 ± 1.0	5.1 ± 0.9	5.0 ± 0.8	4.9 ± 1.0	5.1 ± 1.1	0.78
HDL cholesterol (mmol•L^-1^)	1.3 ± 0.3	1.4 ± 0.3	1.3 ± 0.2	1.3 ± 0.4	1.4 ± 0.3	1.3 ± 0.3	1.2 ± 0.3	0.31
LDL cholesterol (mmol•L^-1^)	3.0 ± 0.8	3.2 ± 0.9	3.5 ± 1.1	3.0 ± 0.9	2.9 ± 0.7	3.0 ± 0.8	3.2 ± 0.9	0.47
Triglycerides (mmol•L^-1^)	1.4 ± 0.6	1.2 ± 0.5	1.4 ± 0.3	1.6 ± 0.7	1.4 ± 0.6	1.4 ± 0.6	1.5 ± 0.7	0.73
Glucose (mmol•L^-1^)	5.4 ± 0.9	5.5 ± 0.5	5.2 ± 0.5	5.5 ± 1.2	5.4 ± 0.9	5.4 ± 0.6	5.1 ± 0.6	0.83
Insulin (pmol•L^-1^)	271 ± 331	252 ± 333	60 ± 50	351 ± 377	274 ± 410	227 ± 195	N/A	0.33
HOMA-IR	9.6 ± 11.4	9.5 ± 13.7	2.0 ± 1.6	12.5 ± 13.4	9.3 ± 12.9	8.1 ± 7.4	N/A	0.28
Cortisol (μg•dL^-1^)	34.9 ± 17.5	38.1 ± 31.8	35.6 ± 20.1	33.4 ± 16.7	34.7 ± 14.7	35.7 ± 16.2	N/A	0.96
Ketones (μM)	0.06 ± 0.04	0.18 ± 0.15	0.09 ± 0.09	0.10 ± 0.13	0.08 ± 0.06	0.08 ± 0.06	N/A	0.29
Leptin (pg•mL^-1^)	21.2 ± 6.4	20.1 ± 6.8	23.0 ± 7.5	22.8 ± 7.9	22.8 ± 7.9	21.2 ± 7.0	N/A	0.76
								
*Dietary Intake*	Grand Mean	HED	ND	VLCHP	LCMP	HCLP	CON	*P*-value

Caloric intake (kcal/kg/day)	21.7 ± 6.8	23.6 ± 5.3	22.3 ± 4.4	20.9 ± 6.4	21.1 ± 5.1	22.4 ± 8.5	---	0.87
Carbohydrate (g/kg/day)	2.5 ± 0.9	2.8 ± 0.9	2.4 ± 0.4	2.4 ± 0.9	2.4 ± 0.7	2.6 ± 1.1	---	0.24
Protein (g/kg/day)	0.9 ± 0.3	0.7 ± 0.2	0.8 ± 0.1	0.9 ± 0.3	0.9 ± 0.4	0.9 ± 0.3	---	< 0.001
Fat (g/kg/day)	0.9 ± 0.3	1.1 ± 0.2	1.1 ± 0.4	0.9 ± 0.2	0.9 ± 0.3	0.9 ± 0.4	---	0.95

All data is presented as means ± SD at baseline. Significance level was set at 0.05. ^d^Different than VLCHP, *P *< 0.001 - 0.05; ^f ^Different than HED, *P *< 0.010. NOTE: No other differences at baseline existed (*P *> 0.05).

### Testing Sessions

Participants were recruited throughout the community through advertisements in local newspapers, campus mailings and television announcements. Those participants interested in participating first contacted the laboratory where they underwent a preliminary screening for the pre-established exclusionary criteria. Eligible participants then completed a familiarization which provided additional education and screening about all aspects of the study protocol. It was during this time that contact information, medical history and informed consent documents were completed. Prior to baseline testing and all other testing sessions, participants were instructed to complete a 4 day food record, observe a 10 h fast and refrain from any vigorous activity for at least 24 h prior to testing. In a standardized fashion to control for natural variations in blood and energy expenditure measures, all reasonable attempts were made to schedule follow-up assessments at a similar time as baseline testing. Baseline testing sessions were identical to the testing sessions after 10 and 14 weeks and consisted of blood collection, anthropometric, resting energy expenditure, aerobic and anaerobic fitness assessments and body composition analysis using DXA. An additional testing session occurred after 1 week of dieting and consisted of all measures at baseline with the exception of aerobic and anaerobic fitness assessments. After baseline testing, participants in all diet and exercise groups, with the exception of the CON group, began weekly 30 min circuit-style resistance training and callisthenic exercise program, which consisted of eight bi-directional exercises, interspersed with callisthenic activities. Under the supervision of fitness instructors, heart rate was monitored during each workout via palpation of the carotid or radial artery in an effort to maintain appropriate exercise intensity.

### Diet Assignments

Phase I of dieting lasted for one week. During this time, participants were assigned to follow the prescribed exercise program and follow one of four diet + exercise combinations (presented as kcals; % carbohydrate: protein: fat): 1) a high energy, high carbohydrate, low protein diet (HED) [2,600; 55:15:30%], 2) a very low carbohydrate, high protein diet (VLCHP) [1,200; 7:63:30%], 3) a low carbohydrate, moderate protein diet (LCMP) [1,200; 20:50:30%], or 4) a high carbohydrate, low protein diet (HCLP) [1,200; 55:15:30%]. Phase II lasted for last for nine weeks, increased caloric intake in all restricted groups to 1,600 calories per day and changed the macronutrient ratios of VLCHP and HCLP to 1,600; 15:55:30%. Further, the HED group altered their macronutrient ratio 2,600; 40:30:30%. Phase III was a four week period which had all participants follow the same diet (2,600; 55:15:30%) and participants were instructed to weigh themselves each day. Upon gaining 3 pounds, participants were told to follow their phase I diet until the additional weight was lost. All subjects were tested over the course of a 14 week period at 0, 1, 10, and 14 weeks to determine any changes in criterion variables. After baseline testing, participants were matched into clusters according to body mass and age into one of six groups and depending on group assignment transitioned through three phases of dieting. Two control groups were used, a no exercise and no diet control group (CON) and an exercise-only control (no diet) group (ND). Phase I dieting lasted for one week where individuals in the high energy, high carbohydrate, low protein diet group (HED) followed a 2,600 kcal•d^-1 ^diet, while those participants following a low-calorie diet consumed a 1,200 kcals•d^-1 ^to stimulate weight loss in addition to providing a determination of the acute (one week) adaptations made relative to energy expenditure and metabolic hormones. These respective diet groups included a very low carbohydrate, high protein diet (VLCHP) [1,200; 7:63:30%], a low carbohydrate, moderate protein diet (LCMP) [1,200; 20:50:30%], and a high carbohydrate, low protein diet (HCLP) [1,200; 55:15:30%]. Phase II dieting lasted nine weeks and incorporated an increase in energy intake to 1,600 kcals•d^-1 ^in those diet groups previously consuming 1,200 kcal•d^-1 ^(e.g., VLCHP, LCHP and HCLP). This slight change was made in an attempt to help promote steady weight loss without stimulating negative perturbations of metabolic factors throughout this nine week phase of dieting. Additionally, slight changes were made to the macronutrient distribution of several diets. The HED group began following a 2,600; 40:30:30% diet and the VLCHP and LCMP groups began following the same 1,200; 55:15:30% diets. These slight changes were made to further explore the impact of varying macronutrient distributions and were made in accordance with our previously published work [3]. No changes were made to the ND, CON and HCLP groups. Over the remaining four weeks (phase III), participants following the low-calorie diets were instructed to follow a 2,600; 55:15:30% diet and record their body mass each day. When participants self-reported a three pound increase in body mass they were instructed to follow their phase I diet until they lost the weight they had gained. Upon losing the weight, they continued to record their body weight each day while returning to the 2,600 kcal•d^-1 ^diet and only began dieting after gaining a total of three pounds. This pattern was continued for the remaining four weeks where all baseline testing measures were completed. In an effort to promote adherence and understanding of all dietary groups, a team of registered dieticians developed menu booklets which outlined examples of each phase of dieting, which included several variations to minimize boredom as well as incorporated substitutions to allow for more flexibility and personal tastes. Throughout the study, familiarization sessions were conducted by the dieticians in addition to being available by phone and during all testing sessions.

### Procedures

#### Anthropometrics

At the beginning of every testing session, subjects had their height and body mass measured according to standard procedures using a Healthometer (Bridgeview, IL) self-calibrating digital scale with an accuracy of ± 0.02 kg. Waist and hip circumference was measured using a Golnick tensiometer using standard ACSM criteria [26]. Resting heart rate was measured via palpation of the radial artery and resting blood pressure was determined using a mercury sphygmomanometer according to previously accepted procedures [26].

#### Dietary Inventories

Prior to each testing session (weeks 0, 1, 10 and 14), subjects were instructed to record all food and fluid intake over a 4 d period, which was reflective of their normal dietary intake and to include one weekend day and three week days. Dietary inventories were then reviewed by a registered dietician and analyzed for average caloric and macronutrient intake using the ESHA Food Processor (Version 8.6) Nutritional Analysis software (Salem, OR) to assess compliance of dietary assignments.

#### Body Composition and Energy Expenditure Assessments

During each testing session, resting energy expenditure assessments were made using a Parvo Medics TrueMax 2400 Metabolic Measurement System (Sandy, UT). This test was a non-exertional test performed in a fasted state and involved the subjects lying supine on an exam table, and having a light blanket placed over them to keep warm. A clear, hard plastic hood and soft, clear plastic drape was then placed over the subjects' neck and head in order to determine resting oxygen uptake and energy expenditure. All participants remained motionless and were instructed to not fall asleep to prevent additional reduction of metabolic rate, for approximately 20 minutes. Data was only recorded after the first ten minutes of testing and throughout a five minute period of time in which criterion variables (e.g., VO_2 _L•min^-1^) changed less than 5% [27]. The reported coefficient of variation in lean, healthy individuals as reported from the manufacturer for this device was ± 2%. Additionally, participants had their bone density and body composition assessed with a whole-body scan using a Hologic Discovery W DXA using software version 12.1 (Waltham, MA). Previous studies have indicated DXA to be an accurate and reliable means to assess changes in body composition [28]. Prior to testing, all participants were properly informed of any inherent risks that could possibly be present from radiation exposure. Briefly, this test involved having the subject lie down on their back in a standardized position in a pair of shorts/t-shirt or a gown. A low dose of radiation was then used to scan their entire body, taking approximately seven minutes, for determination of bone, fat and muscle mass.

#### Blood Collection Procedures

Preceding each testing session, participants were required to fast for 10 h prior to donating approximately four teaspoons (20 milliliters) of blood from an antecubital vein using standard phlebotomy procedures. Two serum separation tubes were immediately centrifuged at 1100 × *g *for 15 min using a standard bench top centrifuge (Cole Palmer, Vernon Hills, IL, Model # 17250-10) prior to having the serum removed and placed into separate micro centrifuge tubes and frozen at -20°C for later analysis of clinical chemistry panels and hormone concentrations. A single lavender top tube containing K_2 _EDTA was used for all whole blood measures. This tube was immediately refrigerated prior to being analyzed on the same day (typically after 4 - 6 h) for a complete blood count with platelet differentials using an Abbott Cell Dyn 3500 (Abbott Laboratories, Abbott Park, IL) automated hematology analyzer (hemoglobin, hematocrit, red blood cell counts, MCV, MCH, MCHC, RDW, white blood cell counts, neutrophils, lymphocytes, monocytes, eosinophils, baosphils). All serum samples were analyzed using a Dade Behring Dimension RXL (Deerfield, IL) automated clinical chemistry analyzer that was calibrated and optimized according to manufacturer guidelines [29]. After centrifugation, each serum sample was assayed for a standard complete metabolic panel (glucose, AST, ALT, GGT, albumin, globulin, sodium, chloride, calcium, carbon dioxide, total bilirubin, alkaline phosphatase), thyroid panel (e.g. T3 (triiodothyronine), T4 (thyroxine), thyroxine uptake, free thyroxine index, and thyroid stimulating hormone), lipid panel (triglycerides, total cholesterol, HDL, LDL, total cholesterol:HDL) and clinical markers of protein and fatty acid metabolism (uric acid, creatinine, BUN, BUN:creatinine ratio, total protein, CK, ketones [betahydroxybutyrate], and LDH). In a follow-up fashion, remaining serum was then assayed using standard commercially available (DS Laboratories, Webster, TX) enzyme-linked immunoabsorbent assays (ELISAs) for leptin (DSL-10-23100), cortisol (DSL-1-2000) and insulin (DSL-10-1600). Prior to analysis, serum samples were diluted 3-fold to get leptin values within the recommended standard curve. Serum concentrations were assessed by determined using a Wallac-Victor IV (Perkin-Elmer Life Sciences, Boston, MA) micro plate reader at an optical density of 450 nm against a known standard curve using standard ELISA procedures according to manufacturer guidelines.

#### Cardiopulmonary Exercise Tests

At baseline and after 10 and 14 weeks of following the diet and exercise program, participants were required to complete a volitional maximal cardiopulmonary exercise test according to the Bruce protocol [30]. Using standard electrode placement and a Quinton 710 ECG unit (Bothell, WA), 12-lead electrocardiogram tests were completed to assess heart function according to previously established criteria [26]. Resting (supine and standing) ECG, heart rate and blood pressure assessments were made prior to commencing the exercise test. After the onset of exercise, ECG, heart rate and blood pressure assessments were taken at the end of every three minute stage, the earliest point after volitional fatigue and after three minutes of active and three minutes of passive recovery. Participants were instructed to perform each test for as long as possible to ensure a true maximal attempt. Standard ACSM test termination criteria were monitored and followed throughout each test [26]. Expired gases (resting and exercise) were collected using a Parvo Medics TrueMax 2400 Metabolic Measurement System (Sandy, UT) using standard procedures and criteria. Accuracy of the oxygen and carbon dioxide analyzers for this device was ± 2%.

#### Maximal Strength and Endurance Assessments

At baseline and after 10 and 14 weeks of following the diet and exercise program, participants performed one-repetition maximum (1RM) assessments and then completed a maximal repetitions to fatigue test using 80% of their pre-determined 1RM with both the bench press and leg press. A warm-up of two sets of 10 repetitions at ~50% 1RM was followed by three to five progressive 1RM attempts with two minutes rest in between attempts using a standard 20 kilogram barbell and a standard bench found in many fitness facilities (Nebula Fitness, OH). Once bench press 1RM was determined, subjects were given five minutes rest and completed a maximal repetitions to fatigue test with 80% of their pre-determined 1RM with the bench press to assess upper-body muscular endurance. Subjects were then given five minutes of rest, and had their maximal muscular strength (1RM) and muscular endurance (80% 1RM repetition to fatigue) determined using a standard hip sled/leg press (Nebula Fitness, OH) using similar testing conditions as the bench press. During all testing sessions, subjects were equally advised using standardized lifting criteria [31-33] and encouraged by the testers. Test to test reliability of performing these strength tests in our lab has yielded low mean coefficients of variation and high reliability for the bench press (CV: 1.9%, intra-class *r *= 0.94) and hip sled/leg press (CV: 0.7%, intra-class *r *= 0.91) [34].

#### Weekly Resistance Training Program

With the exception of the CON group, all participants were randomized to participate in a supervised exercise program three days per week each week throughout the protocol. Each circuit-style workout consisted of eight bi-directional exercises constructed with pneumatic or hydraulic resistance that targeted opposing muscle groups in a concentric-only fashion. Participants were informed of proper use of all equipment and were instructed to complete as many repetitions as they could in a 30 s time period. In an interval fashion, participants performed floor-based callisthenic exercises for a 30 s time period after each resistance exercise in an effort to maintain an exercise heart rate that corresponded to 60% to 80% of their maximum heart rate [26]. All workouts were supervised by trained fitness instructors who assisted with proper exercise technique and maintenance of an adequate exercise heart rate. Participants were required to complete three complete circuits, which corresponded to exercising for approximately 25 minutes followed by a standardized whole-body stretching routine. Attendance was recorded at each workout to monitor compliance to the exercise program.

### Statistical Analysis

Data are presented in all tables and throughout the text as mean ± standard deviation $\left(\bar{\text{X}}\pm \text{SD}\right)$ for the HED, ND, VLCHP, LCHP, HCLP and CON groups, respectively. Week 14 delta values (week 14 - baseline testing) were calculated and used for determination of delta changes across time. Energy expenditure data was first normalized to body mass in kg before being analyzed using 6 × 4 (group × test [0, 1, 10 and 14 weeks]) repeated measures ANOVA with Bonferroni corrections to effectively assess the changes in energy expenditure after the first phase of dieting, which was the most hypo-energetic. All remaining data were analyzed using 6 × 3 (group × test [0, 10 and 14 weeks]) repeated measures ANOVA with Bonferroni corrections. LSD pair-wise comparisons were used to analyze any significant group × time interaction effects. When the sphericity assumption was not met, the conservative Huynh-Feldt correction was used to determine significance level. Pearson product correlations were used to determine any relationship between criterion variables and an alpha level of 0.05 was adopted throughout to prevent any Type I statistical errors.

## Results

### Nutritional Intake and Compliance

All dietary intake was normalized to changes in body mass and average baseline dietary intake is provided in table 2. An inadequate number of dietary records were able to be retrieved from the control (CON) group and as a result were not analyzed. No significant (p = 0.87) group × time interaction effect was found for changes in caloric intake. A main effect for time revealed a significant decrease in caloric intake when compared to baseline after 10 (p < 0.001) and 14 weeks (p < 0.05) of following the protocol. A significant (p < 0.001) group × time interaction effect was revealed for relative protein intake. As expected, pair wise comparisons revealed that protein intake significantly (p < 0.05) increased in the VLCHP and LCMP groups when compared to HCLP. Relative fat intake exhibited no group × time interaction effect (p = 0.95), but a significant main effect for time was yielded (p < 0.05). As expected, reductions in relative fat intake within all dieting groups led to a significant reduction in fat intake after 10 (p < 0.05) and 14 weeks (p < 0.001) of dieting. No significant group × time interaction effect (p = 0.24) was found for relative carbohydrate intake. A significant time effect was found (p < 0.05) whereby carbohydrate intake was reduced after 10 weeks (p < 0.05), but returned to baseline levels after 14 weeks (p = 0.064).

**Table 2:** Anthropometric, body mass, body composition and energy expenditure changes for the high energy, high carbohydrate diet + exercise (HED; 2,600: 55:15:30), no diet + exercise (ND), very low carbohydrate, high protein diet + exercise (VLCHP: 1,200; 63:7:30), low carbohydrate, moderate protein diet + exercise (LCMP: 1,200; 50:20:30), high carbohydrate, low protein diet + exercise (HCLP: 1,200; 55:15:30) and control (CON) groups.

						P-value	
						
Variable	Group	Week 0	Week 1	Week 10	Week 14	Within Group	G × T
Waist (cm)	HED	97.0 ± 12.1	93.3 ± 15.3	94.1 ± 11.8	94.4 ± 12.1	0.78	0.22
	ND	94.4 ± 22.2	93.7 ± 22.5	91.9 ± 21.3	93.7 ± 25.3	0.83	
	VLCHP	99.2 ± 15.4	97.7 ± 13.0	94.7 ± 12.7*	93.6 ± 12.5*^†a^	< 0.001	
	LCMP	101.8 ± 13.5	99.4 ± 13.0	96.1 ± 12.2*	95.6 ± 11.6*^†a^	< 0.001	
	HCLP	97.8 ± 11.0	96.1 ± 10.9	94.4 ± 10.8*	93.6 ± 10.4*	< 0.001	
	CON	92.3 ± 13.2	91.2 ± 14.2	93.7 ± 14.7	93.8 ± 17.9	0.61	

Body Mass (kg)	HED	87.5 ± 16.2	86.9 ± 15.2	85.9 ± 13.1	84.0 ± 11.9^a^	0.26	< 0.005
	ND	92.6 ± 28.4	92.0 ± 27.7	90.5 ± 27.4	90.1 ± 25.5	0.18	
	VLCHP	94.5 ± 18.1	92.7 ± 17.8*	89.8 ± 17.6*^†^	89.5 ± 18.0*^†a^	< 0.001	
	LCMP	95.1 ± 14.9	93.8 ± 14.8*	91.6 ± 14.3*^†^	91.4 ± 14.1*^†a^	< 0.001	
	HCLP	93.1 ± 15.0	91.8 ± 14.9*	89.2 ± 15.5*^†^	89.5 ± 14.6*^†a^	< 0.001	
	CON	93.2 ± 16.6	85.8 ± 16.6	87.0 ± 17.4	86.8 ± 18.4	0.42	

DXA Fat-Free Mass (kg)	HED	46.3 ± 7.9	46.4 ± 7.6	46.6 ± 7.3	44.9 ± 6.3^a^	0.13	< 0.05
	ND	47.3 ± 10.8	47.5 ± 10.7	46.7 ± 10.4	47.5 ± 9.2	0.66	
	VLCHP	48.6 ± 7.8	47.8 ± 7.6*	47.7 ± 7.1	47.8 ± 7.8	< 0.05	
	LCMP	47.0 ± 5.6	46.6 ± 5.5	46.7 ± 5.8	47.0 ± 6.0	0.26	
	HCLP	47.6 ± 6.0	47.8 ± 5.9*	46.8 ± 5.9	46.8 ± 5.8*	< 0.05	
	CON	45.0 ± 7.1	45.1 ± 7.3	45.8 ± 7.3	45.6 ± 8.4	0.18	

DXA Fat Mass (kg)	HED	35.0 ± 9.0	34.7 ± 8.7	33.4 ± 6.8	33.3 ± 6.7	0.27	< 0.05
	ND	39.4 ± 16.4	38.8 ± 16.6	37.4 ± 15.0	36.4 ± 14.8	0.08	
	VLCHP	39.2 ± 10.3	38.5 ± 10.2*	35.7 ± 10.7*^†^	35.5 ± 10.6*^† a^	< 0.001	
	LCMP	41.8 ± 10.0	40.8 ± 9.9*	38.7 ± 9.5*^†^	38.3 ± 8.8*^† a^	< 0.001	
	HCLP	39.1 ± 9.4	38.5 ± 9.3*	36.4 ± 9.3*^†^	36.1 ± 9.2*^† a^	< 0.001	
	CON	34.7 ± 8.9	34.3 ± 8.6	34.7 ± 9.8	34.7 ± 9.7	0.74	

DXA % Fat (%)	HED	42.7 ± 4.6	42.4 ± 4.8	41.6 ± 3.6	42.4 ± 4.3	0.19	0.09
	ND	44.5 ± 4.4	44.0 ± 4.2	43.7 ± 3.5	42.5 ± 4.8	0.22	
	VLCHP	44.2 ± 4.3	44.1 ± 4.4	42.1 ± 5.0*^†^	42.0 ± 5.1*^†e^	< 0.001	
	LCMP	46.5 ± 4.6	46.2 ± 4.8	44.9 ± 4.9*^†^	44.6 ± 4.6*^†e^	< 0.001	
	HCLP	44.6 ± 3.8	44.6 ± 3.9	43.1 ± 4.3*^†^	43.0 ± 4.4*^†^	< 0.001	
	CON	43.1 ± 2.6	42.8 ± 2.3	42.3 ± 2.6	42.1 ± 2.2	0.08	

REE (kcals•d^-1^)	HED	1297 ± 319	1469 ± 260	1391 ± 255	1508 ± 205	0.08	0.08
	ND	1462 ± 246	1571 ± 307	1640 ± 269	1585 ± 282	0.19	
	VLCHP	1690 ± 283	1606 ± 318*	1649 ± 296	1708 ± 298^†e^	< 0.01	
	LCMP	1642 ± 249	1570 ± 222	1616 ± 337	1639 ± 246^e^	0.18	
	HCLP	1642 ± 247	1572 ± 266	1648 ± 262^†^	1659 ± 228^†e^	< 0.01	
	CON	1720 ± 265	1621 ± 281	1708 ± 259	1656 ± 254^e^	0.62	

Dietary intake is presented as calorie intake: % carbohydrate: protein: fat. All data is presented as group means ± standard deviation for week 0, week 1, week 10 and week 14. Individual main effects for time are provided as within-group *P*-values. Group × time interaction effects are provided as GxT *P*-values. Between-group significance indicators represent the delta change from baseline at week 14. Significance level was 0.05. *Different from baseline, *P *< 0.05, ^†^Different than week 1, *P *< 0.05, ^a^Different than CON, *P *< 0.05, ^e^Different from HED, *P *< 0.05.

### Anthropometric Changes

**Figure 2:**
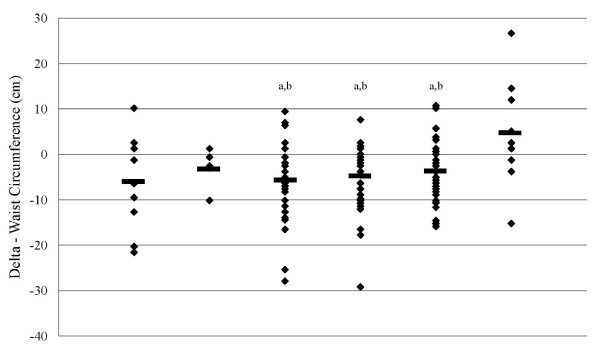
**Delta change in waist circumference (cm) at 14 weeks**. Data are presented as individual changes from baseline. HED = high-energy, high carbohydrate diet + exercise (n = 9); ND = no diet + exercise (n = 5); VLCHP = very low carbohydrate, high protein diet + exercise (n = 39); LCMP = Low carbohydrate, moderate protein + exercise (n = 36); HCLP = High carbohydrate, low protein + exercise (n = 43); CON = no diet + no exercise (n = 9). ^a^Different than CON (p < 0.05), ^b^Different from ND (p < 0.05).

**Figure 3:**
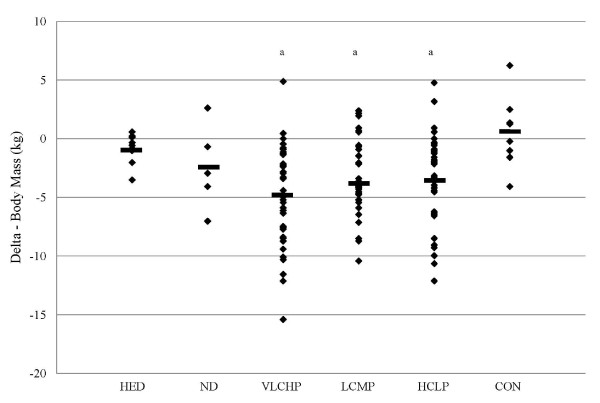
**Delta change in body mass (kg) at 14 weeks**. Data are presented as individual changes from baseline. HED = high-energy, high carbohydrate diet + exercise (n = 9); ND = no diet + exercise (n = 5); VLCHP = very low carbohydrate, high protein diet + exercise (n = 39); LCMP = Low carbohydrate, moderate protein + exercise (n = 36); HCLP = High carbohydrate, low protein + exercise (n = 43); CON = no diet + no exercise (n = 9). ^a^Different than CON (p < 0.05).

Anthropometric measurements which included body mass and waist circumference were recorded after 0, 1, 10 and 14 weeks. Significant main effects for time (p < 0.001) but no significant group × time interaction effect (p = 0.22) in waist circumference was revealed when all groups were analyzed together. Restriction of energy intake while exercising irrespective of macronutrient distribution (VLCHP, LCMP and HCLP) resulted in a significant reduction in waist circumference after 10 weeks (VLCHP: -4.5 ± 8.2 cm; p < 0.001, LCMP: -5.7 ± 6.6 cm; p < 0.001 and HCLP: -3.4 ± 7.2 cm; p < 0.001) and continued until 14 weeks (VLCHP: -5.6 ± 9.2 cm; p < 0.001, LCMP: -6.2 ± 7.3 cm; p < 0.001 and HCLP: -4.2 ± 6.5 cm; p < 0.001) (Figure 2). No changes (p > 0.05) were seen at either of these time points for HED, ND and CON (See table 2). Significant between-group changes occurred after 14 weeks in VLCHP (-5.6 ± 9.2 cm; p < 0.05) and LCMP (-6.2 ± 7.3 cm; p < 0.05) when compared to CON (1.6 ± 11.1 cm), but no other groups (Table 2). When evaluating changes in body mass, a significant main effect over time (p < 0.001) in addition to a significant group × time interaction effect (p < 0.005) was observed. Changes in body mass also revealed that those groups which restricted their caloric intake while exercising, irrespective of macronutrient distribution, experienced significant reductions in body mass after 1 week (VLCHP: -1.8 ± 1.1 kg; p < 0.001, LCMP: -1.3 ± 1.5 kg; p < 0.001 and HCLP: -1.3 ± 1.0 kg; p < 0.001) and experienced further reductions from this time point after 10 weeks of following the program (VLCHP: -4.7 ± 3.2 kg; p < 0.001, LCMP: -3.6 ± 2.9 kg; p < 0.001 and HCLP: -3.9 ± 4.5 kg; p < 0.001) (Figure 3). Furthermore, changes in body mass in all restricted energy groups after 14 weeks were significantly reduced when compared to baseline and week 1, but were not different than week 10 (Table 2). These changes after 14 weeks resulted in significantly greater amounts of body mass loss for all caloric restriction groups (VLCHP: -5.0 ± 4,2 kg; p < 0.001, LCMP: -3.7 ± 3.3 kg; p < 0.01 and HCLP: -3.6 ± 3.8 kg; p < 0.01) when compared to CON (0.5 ± ± 2.9 kg).

### Body Composition

**Figure 4:**
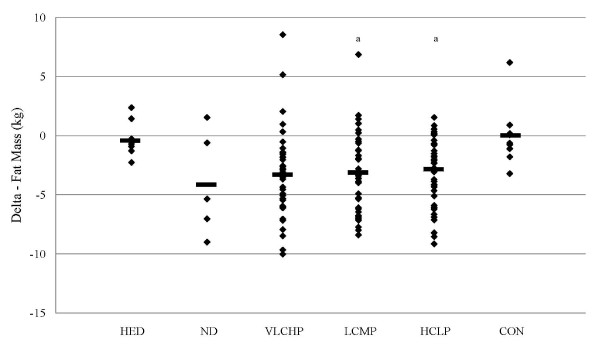
**Delta change in DXA fat mass (kg) at 14 weeks**. Data are presented as individual changes from baseline. HED = high-energy, high carbohydrate diet + exercise (n = 9); ND = no diet + exercise (n = 5); VLCHP = very low carbohydrate, high protein diet + exercise (n = 39); LCMP = Low carbohydrate, moderate protein + exercise (n = 36); HCLP = High carbohydrate, low protein + exercise (n = 43); CON = no diet + no exercise (n = 9). ^a^Different than CON (p < 0.05).

Body composition was assessed using dual-energy x-ray absorptiometry after 0, 1, 10 and 14 weeks (Table 2). A significant group × time interaction effect (p < 0.05) for DXA fat-free mass was reported. Within group changes over time that all energy restricted group experienced reductions in DXA fat-free mass, but only those changes in VLCHP and HCLP were significant. Furthermore, pair wise comparisons of this effect revealed that significant reductions in fat-free mass in VLCHP only occurred after phase I dieting (-0.8 ± 1.3 kg; p < 0.005) as DXA fat-free mass values tended to be different after 10 weeks (-0.9 ± 2.1 kg; p = 0.07) and returned to baseline values after 14 weeks. HCLP changes in DXA fat-free mass were significant after week 1 (-0.8 ± 1.3 kg; p < 0.005) and week 10 (-0.7 ± 1.5 kg; p < 0.05) and tended to be reduced after 14 weeks (-0.8 ± 1.8 kg; p = 0.051). Interestingly, HED experienced a reduction in DXA fat-free mass after 14 weeks (-1.5 ± 3.2 kg; p < 0.05) that was significantly different than the changes seen for CON (0.6 ± 1.5) at this time point. DXA fat mass experienced a significant main effect over time (p < 0.001) and a significant group × time interaction effect (p < 0.05). Significant within group changes were again seen in all groups that restricted their caloric intake (e.g., VLCHP, LCMP, HCLP). Significant reductions in fat mass were seen after 1 week (VLCHP: -0.7 ± 1.0 kg; p < 0.001, LCMP: -0.9 ± 1.2 kg; p < 0.001 and HCLP: -0.6 ± 1.3 kg; p < 0.001) (Figure 4). Even further reductions were then reported after 10 weeks (VLCHP: -3.6 ± 2.1 kg; p < 0.001, LCMP: -3.0 ± 2.6 kg; p < 0.001 and HCLP: -2.7 ± 2.6 kg; p < 0.001) which were maintained after 14 weeks (VLCHP: - 3.7 ± 2.9 kg; p < 0.001, LCMP: -3.5 ± 4.7 kg; p < 0.001 and HCLP: -2.9 ± 2.9 kg; p < 0.001) of following their respective diet and exercise program (Table 2).

Similarly, DXA % fat values experienced a significant main effect over time (p < 0.001) while the group × time interaction effect approached significance (p = 0.09). Significant within group changes in VLCHP, LCMP, and HCLP were found after 10 weeks (VLCHP: -2.1 ± 1.8%; p < 0.001, LCMP: -1.7 ± 2.1%; p < 0.001 and HCLP: -1.5 ± 1.9%; p < 0.001) which were maintained after 14 weeks (VLCHP: -2.2 ± 1.8%; p < 0.001, LCMP: -1.9 ± 2.0%; p < 0.001 and HCLP: -1.6 ± 2.2%; p < 0.001). Furthermore, these changes resulted in significantly greater reductions in DXA % fat in VLCHP and HCLP groups when compared to the CON group.

### Energy Expenditure

**Figure 5:**
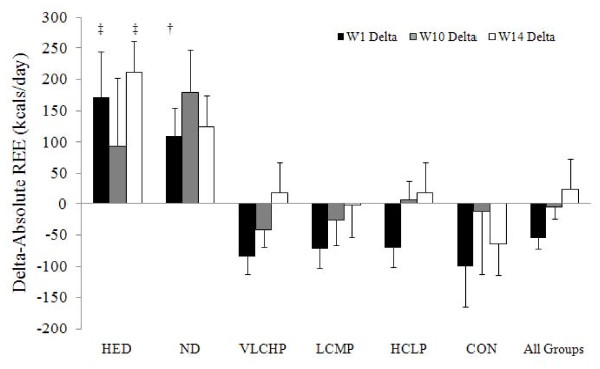
**Delta change in absolute resting energy expenditure (kcals•d^-1^) at 0, 10 and 14 weeks HED = high-energy, high carbohydrate diet + exercise (n = 9); ND = no diet + exercise (n = 5); VLCHP = very low carbohydrate, high protein diet + exercise (n = 39); LCMP = Low carbohydrate, moderate protein + exercise (n = 36); HCLP = High carbohydrate, low protein + exercise (n = 43); CON = no diet + no exercise (n = 9)**. ‡Different from all groups except ND (p < 0.05), †Different from all three diet groups (e.g., VLCHP, LCMP, HCLP) (p < 0.05).

Fasting resting energy expenditure measurements (REE) were obtained at 0, 1, 10 and 14 weeks (Table 2 and Figure 5). Raw data (kcal•d^-1^) is provided in table 2 while all statistical analysis was completed using relative resting energy expenditure data (kcal•kg•d^-1^). A significant main effect for time (p < 0.001) was reported along with a significant group × time interaction effect (p < 0.01). After phase I dieting, non-significant (p > 0.05) reductions occurred in all energy restricted groups and CON. After 10 and 14 weeks, respectively, all groups (irrespective of dieting status) reported greater REE values from baseline with the exception of CON. As expected, the HED group experienced the greatest increase after 14 weeks in relative REE (3.3 ± 1.8 (kcal•kg•d^-1^); a change that was significantly greater than VLCHP (1.1 ± 2.1 kcal•kg•d^-1^; p < 0.005), LCMP (0.6 ± 2.0 kcal•kg•d^-1^; p < 0.001), HCLP (0.9 ± 2.2 kcal•kg•d^-1^; p < 0.005) and CON (-0.8 ± 1.8 kcal•kg•d^-1^; p < 0.001).

### Fitness Changes

Maximal cardiopulmonary, muscular strength and muscular endurance assessments were taken after 0, 10 and 14 weeks (Table 3). As expected, all groups that restricted energy intake and followed the exercise program experienced similar significant increases (p < 0.05) in relative maximal VO_2 _value (8.8 ± 13.7%), relative bench press 1RM (16.5 ± 17.8%) and relative leg press 1RM (16.2 ± 16.4%) compared to control group values of (-0.8 ± 7.4%, 6.5 ± 13.9% and 8.3 ± 17.8%), respectively. Significant main effects for time (p < 0.005) and group × time interaction effects (p < 0.05) were reported for maximal aerobic capacity data. Significant increases in VO_2_Max were reported after 10 weeks for VLCHP (2.3 ± 2.7 ml/kg/min; p < 0.001) and HCLP (2.0 ± 2.0 ml/kg/min; p < 0.001); which both remained significantly greater (p < 0.005) than baseline after 14 weeks. Aerobic fitness improved in LCMP, but these changes were not significant until after 14 weeks (1.5 ± 2.3 ml/kg/min; p < 0.005). When compared to CON (-0.2 ± 1.9 ml/kg/min), VLCHP (2.0 ± 3.5 ml/kg/min; p < 0.05) and HCLP (1.8 ± 1.9 ml/kg/min; p < 0.05) groups experienced significantly greater increases in aerobic fitness after 14 weeks. Interestingly and after 14 weeks of exercise training, the HCLP group (4.1 ± 6.3 kg•kg^-1^) exhibited significantly greater upper-body strength when compared to VLCHP (1.9 ± 4.9 kg•kg^-1^; p < 0.05). Similarly, HCLP lower-body strength changes (15.5 ± 27.6 kg•kg^-1^) were significantly different than VLCHP (10.1 ± 25.0 kg•kg^-1^; p < 0.01) and LCMP (12.3 ± 19.2 kg•kg^-1^; p < 0.05). While the lifting volume completed for each group increased as a result of training, none of these changes were significant.

**Table 3:** Cardio respiratory and muscular fitness changes for the high energy, high carbohydrate diet + exercise (HED; 2,600: 55:15:30), no diet + exercise (ND), very low carbohydrate, high protein diet + exercise (VLCHP: 1,200; 63:7:30), low carbohydrate, moderate protein diet + exercise (LCMP: 1,200; 50:20:30), high carbohydrate, low protein diet + exercise (HCLP: 1,200; 55:15:30).

					P-value	
					
Variable	Group	Week 0	Week 10	Week 14	Within Group	G × T
Max VO_2 _(mL•kg•min^-1^)	HED	23.5 ± 3.2	22.9 ± 4.6	24.6 ± 3.9	0.22	< 0.05
	ND	21.4 ± 4.1	22.2 ± 4.0	21.9 ± 3.5	0.45	
	VLCHP	21.6 ± 4.0	23.9 ± 4.3*	23.5 ± 4.4* ^a^	< 0.001	
	LCMP	21.3 ± 4.6	22.0 ± 5.2	22.8 ± 4.7*	< 0.05	
	HCLP	21.7 ± 3.4	23.8 ± 4.4*	23.5 ± 3.9*^a^	< 0.001	
	CON	23.7 ± 4.9	24.0 ± 5.6	23.5 ± 4.9	0.80	

BP 1RM (kg•kg^-1^)	HED	0.41 ± 0.09	0.46 ± 0.08	0.49 ± 0.08*	< 0.001	0.39
	ND	0.36 ± 0.07	0.39 ± 0.05	0.42 ± 0.09*	0.053	
	VLCHP	0.39 ± 0.13	0.43 ± 0.12*	0.43 ± 0.12*^f^	< 0.001	
	LCMP	0.36 ± 0.09	0.40 ± 0.09*	0.41 ± 0.09*	< 0.001	
	HCLP	0.36 ± 0.08	0.41 ± 0.09*	0.42 ± 0.09*	< 0.001	
	CON	0.35 ± 0.09	0.37 ± 0.10	0.37 ± 0.09	0.36	

BP Lifting Volume (kg•kg^-1^)	HED	5.1 ± 1.8	5.1 ± 3.7	5.2 ± 4.5	0.98	0.91
	ND	5.5 ± 2.7	5.3 ± 1.9	4.5 ± 1.7	0.64	
	VLCHP	4.2 ± 2.0	4.8 ± 3.1	4.5 ± 2.7	0.30	
	LCMP	4.6 ± 2.3	4.9 ± 1.9	4.7 ± 2.0	0.75	
	HCLP	5.2 ± 2.5	5.2 ± 2.3	5.1 ± 2.2	0.87	
	CON	4.1 ± 2.4	5.2 ± 2.0	5.2 ± 1.4	0.33	

LP 1RM (kg•kg^-1^)	HED	2.1 ± 0.5	2.3 ± 0.6	2.3 ± 0.6	0.10	0.24
	ND	1.6 ± 0.3	1.7 ± 0.2	1.8 ± 0.3	0.08	
	VLCHP	1.9 ± 0.4	2.0 ± 0.5*	2.1 ± 0.5*^f^	< 0.001	
	LCMP	1.8 ± 0.5	1.9 ± 0.5*	2.0 ± 0.5*^f^	< 0.001	
	HCLP	1.7 ± 0.5	2.0 ± 0.5*	2.1 ± 0.5*	< 0.001	
	CON	1.9 ± 0.5	1.9 ± 0.3	2.0 ± 0.4	0.63	

LP Lifting Volume (kg•kg^-1^)	HED	49 ± 30	59 ± 42	56 ± 60	0.61	0.53
	ND	45 ± 19	56 ± 32	38 ± 20	0.48	
	VLCHP	47 ± 26	42 ± 21	47 ± 19	0.29	
	LCMP	42 ± 17	48 ± 24	47 ± 24	0.25	
	HCLP	45 ± 21	48 ± 34	46 ± 25	0.64	
	CON	41 ± 12	43 ± 19	46 ± 21	0.47	

Dietary intake is presented as calorie intake: % carbohydrate: protein: fat. All data is presented as group means ± standard deviation for week 0, week 10 and week 14. Individual main effects for time are provided as within-group *P*-values. Group × time interaction effects are provided as GxT *P *-values. Between-group significance indicators represent the delta change from baseline at week 14. Significance level was 0.05. *Different from baseline, *P *< 0.05, ^a^Different than CON, *P *< 0.05, ^f^Different than HCLP, *P *< 0.05.

### Lipid Panels

No significant changes in total (p = 0.67), HDL (p = 0.90) and LDL cholesterol (p = 0.63) as well as serum levels of triglycerides (p = 0.95) were found for all diet and exercise groups at all time points throughout the investigation (Table 4). No group × time interaction effects (p > 0.05) were reported. All data can be found in table 4.

**Table 4:** Lipid panel, glucose and selected markers of fuel status and utilization for the high energy, high carbohydrate diet + exercise (HED; 2,600: 55:15:30), no diet + exercise (ND), very low carbohydrate, high protein diet + exercise (VLCHP: 1,200; 63:7:30), low carbohydrate, moderate protein diet + exercise (LCMP: 1,200; 50:20:30), high carbohydrate, low protein diet + exercise (HCLP: 1,200; 55:15:30).

					P-value	
					
Variable	Group	Week 0	Week 10	Week 14	**Within **Group	G × T
Total Cholesterol (mmol•L^-1^)	HED	5.2 ± 1.0	4.8 ± 0.9*	5.1 ± 1.3	0.12	0.67
	ND	5.4 ± 1.0	5.1 ± 1.5	5.0 ± 1.0	0.33	
	VLCHP	5.2 ± 1.0	5.2 ± 1.1	5.1 ± 1.2	0.84	
	LCMP	4.9 ± 1.0	4.7 ± 0.9	5.0 ± 0.7	0.11	
	HCLP	4.9 ± 0.9	4.6 ± 0.9	4.7 ± 0.8	0.11	
	CON	5.1 ± 1.1	4.6 ± 1.1	4.9 ± 1.1	< 0.01	

HDL Cholesterol (mmol•L^-1^)	HED	1.4 ± 0.3	1.3 ± 0.2	1.4 ± 0.2	0.16	0.90
	ND	1.3 ± 0.2	1.3 ± 0.2	1.3 ± 0.2	0.55	
	VLCHP	1.3 ± 0.4	1.3 ± 0.3	1.3 ± 0.4	0.94	
	LCMP	1.4 ± 0.4	1.3 ± 0.3	1.4 ± 0.3	0.18	
	HCLP	1.3 ± 0.2	1.3 ± 0.3	1.3 ± 0.3	0.86	
	CON	1.2 ± 0.3	1.2 ± 0.2	1.2 ± 0.3	0.81	

LDL Cholesterol (mmol•L^-1^)	HED	3.2 ± 0.9	2.9 ± 0.7	3.2 ± 1.1	0.10	0.63
	ND	3.5 ± 1.1	3.3 ± 1.6	3.3 ± 1.2	0.55	
	VLCHP	3.1 ± 0.8	3.2 ± 0.9	3.1 ± 1.0	0.97	
	LCMP	2.8 ± 0.7	2.7 ± 0.7	2.9 ± 0.6	0.16	
	HCLP	3.0 ± 0.7	2.8 ± 0.8	2.9 ± 0.7	0.28	
	CON	3.2 ± 0.9	2.8 ± 1.0	3.0 ± 0.8	0.07	

Triglycerides (mmol•L^-1^)	HED	1.2 ± 0.5	1.2 ± 0.4	1.2 ± 0.6	0.89	0.95
	ND	1.4 ± 0.3	1.0 ± 0.3*	1.0 ± 0.2	0.07	
	VLCHP	1.6 ± 0.7	1.4 ± 0.9	1.5 ± 0.9	0.09	
	LCMP	1.4 ± 0.7	1.3 ± 0.6	1.3 ± 0.5	0.10	
	HCLP	1.3 ± 0.6	1.2 ± 0.5*	1.2 ± 0.5	< 0.05	
	CON	1.5 ± 0.7	1.3 ± 0.8	1.5 ± 0.9	0.80	

Insulin (pmol•L^-1^)	HED	252 ± 333	211 ± 312	206 ± 313	0.08	0.46
	ND	60 ± 50	53 ± 46	154 ± 226	0.43	
	VLCHP	351 ± 377	292 ± 341*	294 ± 325*^b^	< 0.005	
	LCMP	274 ± 410	233 ± 392	245 ± 398	0.23	
	HCLP	227 ± 19	187 ± 200	200 ± 218	0.13	
	CON					

Glucose (mmol•L^-1^)	HED	5.5 ± 0.5	5.8 ± 0.5	5.6 ± 0.5	0.20	0.85
	ND	5.2 ± 0.5	5.3 ± 1.3	5.1 ± 0.5	0.86	
	VLCHP	5.5 ± 1.3	5.7 ± 1.9	5.6 ± 1.9	0.33	
	LCMP	5.4 ± 1.0	5.4 ± 0.6	5.3 ± 0.6	0.39	
	HCLP	5.4 ± 0.8	5.4 ± 0.6	5.2 ± 0.6	< 0.05	
	CON	5.1 ± 0.6	5.0 ± 0.6	4.9 ± 0.9	0.75	

HOMA-IR	HED	9.5 ± 13.7	8.1 ± 11.9	7.3 ± 10.6	0.11	0.55
	ND	2.0 ± 1.6	1.7 ± 1.3	5.3 ± 7.8	0.42	
	VLCHP	12.5 ± 13.4	10.9 ± 12.5	10.5 ± 11.6* ^b^	< 0.05	
	LCMP	9.3 ± 12.9	8.0 ± 12.6	7.8 ± 10.9	0.21	
	HCLP	8.1 ± 7.4	6.6 ± 7.4	6.8 ± 7.6	0.11	
	CON					

Leptin (pg•mL^-1^)	HED	21.2 ± 6.4	19.4 ± 4.0	18.6 ± 5.9	0.33	< 0.05
	ND	20.1 ± 6.8	15.4 ± 8.8	19.1 ± 7.9	0.18	
	VLCHP	23.0 ± 7.5	17.1 ± 7.4*	18.0 ± 7.2*	< 0.001	
	LCMP	22.8 ± 7.9	21.8 ± 8.3	20.1 ± 7.8*	< 0.05	
	HCLP	21.2 ± 7.0	17.8 ± 7.0*	17.8 ± 7.7*	< 0.001	
	CON					

Ketones (uM)	HED	0.06 ± 0.04	0.06 ± 0.04	0.16 ± 0.25	0.23	< 0.05
	ND	0.18 ± 0.15	0.21 ± 0.15	0.37 ± 0.64	0.50	
	VLCHP	0.09 ± 0.09	0.14 ± 0.19	0.08 ± 0.06	0.13	
	LCMP	0.10 ± 0.13	0.10 ± 0.11	0.07 ± 0.05	0.34	
	HCLP	0.08 ± 0.06	0.10 ± 0.07	0.07 ± 0.05	0.06	
	CON					

Cortisol (nmol•L^-1^)	HED	1051 ± 878	1049 ± 692	833 ± 353	0.48	0.75
	ND	982 ± 556	1039 ± 485	855 ± 366	0.53	
	VLCHP	921 ± 462	1011 ± 557	954 ± 439^b, e^	0.37	
	LCMP	957 ± 405	939 ± 370	914 ± 413^b, e^	0.79	
	HCLP	986 ± 447	949 ± 446	945 ± 487^b^	0.82	
	CON					

Dietary intake is presented as calorie intake: % carbohydrate: protein: fat. All data is presented as group means ± standard deviation for week 0, week 10 and week 14. Individual main effects for time are provided as within-group *P*-values. Group × time interaction effects are provided as G × T *P*-values. Between-group significance indicators represent the delta change from baseline at week 14. Significance level was 0.05. NOTE: Insulin, HOMA-IR, leptin, ketones and cortisol were not run on the CON group due to an inability to collect an appropriate numbers of samples. *Different from baseline, *P *< 0.05, ^b^Different from ND, *P *< 0.05, ^e^Different from HED, *P *< 0.05.

### Markers of Fuel Utilization and Energy Regulation

The serum samples collected at weeks 0, 10 and 14 weeks were used to assay hormones and selected substrates which were attributed to substrate utilization and energy regulation. In relation to fuel utilization, serum levels of glucose, insulin, cortisol and ketones were measured in addition calculating the homeostatic model assessment for insulin resistance (HOMA-IR) [35]. Serum levels of the adipokine, leptin, were determined as an assessment of appetite regulation at 0, 10 and 14 weeks. We were unable to collect an adequate sample from participants in our control group and as a result no insulin, HOMA-IR, leptin, ketones and cortisol data are available for this group (See Table 4). Overall no significant group × time interaction effects were seen for insulin (p = 0.46), glucose (p = 0.85) and HOMA-IR (p = 0.55). A significant within-group reduction for insulin did occur in the VLCHP group with the 10 week and 14 week values being significantly reduced from their respective baseline values. In conjunction, these changes also led to a significant reduction in HOMA-IR for the VLCHP group at both the 10 week and 14 week time points. A significant main effect for time (p < 0.001) and group × time interaction effect for leptin was observed (p < 0.05). Significant reductions (from baseline) after 10 and 14 weeks, respectively occurred in VLCHP and HCLP while LCMP was significantly reduced after 14 weeks (table 4). No other significant (p > 0.05) main effect or group × time interactive effects were found for all other variables.

## Discussion

The purpose of this study was to study how combinations of a regular exercise and diet program can impact anthropometric and health-related outcomes. Additionally and as we previously attempted [3] we sought to further examine how replacing dietary carbohydrate with protein to varying degrees may impact anthropometric and health outcomes in sedentary, obese women who participated in a regular diet and exercise program. This study represents the second in a series of studies our research group has conducted in an attempt to better understand how various dietary regimens interact with an exercise program and what outcomes can be expected. Currently, the Curves for Women program is followed at over 10,000 fitness franchises across the globe with millions of women worldwide following the program [25]. The study design was based largely upon our previously published study with slight modification in phase I dieting, which was decreased to one week in the present investigation in addition to changes to the weight maintenance period (Phase III). Similar to our previous study, this employed study design has certain strengths such as the overall number of subjects, incorporation of adequate control groups, dietary oversight, and exercise supervision. A potential area of weakness is the unbalanced distribution of participants across all groups which took place as part of study design. The authors acknowledge that increasing (and/or equating) sample size in every group would have addressed this concern and would have been helpful, the primary objective of our study was to determine the impact of different macronutrient distributions and how this alteration impacted the end points measured in the present study. In this respect, however, statistical power analysis of our primary and secondary outcomes revealed power values that ranged from 0.767 - 0.944 and partial eta squared values which ranged from 0.055 - 0.092; providing evidence of appropriate statistical power on our primary and secondary outcomes. We initially hypothesized that following a diet and exercise program would improve all primary and secondary outcomes (waist circumferences and other anthropometric and body composition variables) in comparison to the control groups (no diet + no exercise [CON] and no diet + exercise [ND]). We also hypothesized that those groups ingesting a higher proportion of dietary protein (VLCHP and LCMP) would experience improved responses in our primary and secondary outcomes. The primary findings from this study confirmed our initial hypothesis that following any form of caloric restriction along with the exercise program led to greater improvements in waist circumference (primary) and other secondary outcomes (body mass, DXA fat mass, DXA % fat, resting energy expenditure) in addition to improvements in fitness parameters. This finding also provides continued support that exercise participation alone cannot adequately promote improvements in these end points, a conclusion we [3] and others have previously made [36].

A critical component to our study design and investigated questions was the compliance to the recommended dietary regimens prescribed to all groups. As expected, significant changes did occur for energy, carbohydrate and protein intake between diet phases (e.g., Phase I, Phase II, etc.) and dietary groups (e.g., VLCHP and LCMP vs. HCLP). From an overall standpoint, compliance to the dietary regimens was somewhat successful, but some deviations did result in an inability to make all a priori comparison. In this respect, the HED group was unable to achieve the prescribed energy intake of 2,600 kcals•^-1^; a response which we reported in our initial investigation [3]. Their mean intake of 1,800 kcals•d^-1 ^was approximately 800 kcals•d^-1 ^lower than prescribed, but was still 320 kcals•d^-1 ^greater than the average intake of the other restricted diet groups (e.g., VLCHP, LCMP, HCLP). The higher value still allowed for comparisons between caloric intakes while exercising, which was the main reason for this dietary prescription, even though the associated adaptations we hoped to compare likely did not occur to the extent we planned. The 30% error rate commonly associated with dietary reporting may have contributed to this, and in addition previous reports have suggested that greater errors occur when higher caloric intakes are prescribed. It is also likely that the relatively low fat intake at such a high caloric intake resulted in an overall higher volume of food that was prohibitive versus ingesting a lower volume of higher energy density foods common in the North American diet. Caloric intake, however, was reduced overall by an average of 504 kcals•d^-1 ^in the diet groups (e.g., VLCHP, LCMP, HCLP) from their baseline intakes, which was deemed as a positive response to the prescribed dietary regimens. Lastly and an important point we reported before relates to the relative daily intake of protein for the VLCHP and LCMP groups and inherent safety or risks of this regimen. While both groups ingested a much greater proportion of calories as protein (~50 - 60% daily energy from protein), their relative daily intake of protein (~1.1 gram protein•kg•d^-1^) was not much greater than RDA guidelines. This result is consistent with our previous work [3], but is a lower overall protein intake than what other investigators have employed in similar study designs [6].

Waist circumference was chosen as a primary outcome due to its ability to serve as a predictor of diabetes, cardiovascular disease and other comorbidities [37]. As previously reported, results from this study reveal that regular participation in a resistance-based exercise program, when combined with energy restriction, significantly reduces waist circumference [3]. Independent of macronutrient distribution, reductions in waist circumference were significant over time and greater than CON (Figure 2). Similarly, body mass was also found to significantly decrease in those groups that followed the exercise program and restricted energy intake (Figure 3). No between-group differences were found for those groups that replaced more carbohydrate with protein (VLCHP and LCMP vs. HCLP), although slightly greater decreases in waist circumference and body mass did occur (Table 2); an outcome identical to our previous report [3]. Overall these findings provide support for previous studies in diabetic and non-diabetic populations which also investigated the impact of macronutrient content on changes in weight loss [9,38,39]. While none of the studies incorporated an exercise program, our findings refute conclusions made by Layman et al. who suggested that greater total weight loss occurs, independent of exercise participation when increased dietary protein intake occurs [22]. While greater changes did occur in those groups that ingested more protein in the present, the changes were not statistically significant and thus can't be considered real effects. Furthermore, those participants who restricted energy intake and exercise all lost significant amounts of fat mass (Figure 4) and percent fat (Table 2), but again no between-group differences were evident. It is particular interesting to note that only the HCLP group experienced a significant reduction in DXA fat-free mass after completion of the 14-week program. This decrease resulted in a change in DXA fat-free mass that was significantly different than changes in CON after 14 weeks, but the changes were not different than any other groups in the study. At some level, this finding supports our second hypothesis that a greater intake of dietary protein would spare losses of fat-free mass while restricting energy intake. It has been reported that higher intakes of dietary protein during dieting can prevent losses of lean mass, however, these findings are somewhat mixed [9,22]. Careful interpretation of these data is encouraged as the discussed changes are only within the HCLP and are not a between-group finding. Nonetheless, these findings do suggest that significant amounts of fat can be lost in comparison to changes in fat-free mass while dieting and exercising [5,21], and to a greater extent than control conditions.

An untoward metabolic response to energy restriction is a decrease in energy expenditure [3,38,40]. Our first investigation had participants restrict energy intake to 1,200 kcals•d^-1 ^for two weeks, which resulted in a significant reduction in energy expenditure [3]. To minimize this counteractive response, dieters in the present study restricted calories to 1,200 kcals•d^-1 ^for only week in the present study. While all groups who followed a diet reported a reduction in energy expenditure, only the changes in VLCHP were significant after one week (~84 kcals•d^-1^). Regular participation in the exercise program for 9 weeks returned these values to baseline and in two groups (VLCHP and HCLP) a significant increase occurred in comparison to week one values (Figure 5). Mixed outcomes have been reported for the influence of an exercise program to stimulate energy expenditure values [22,41]. Irrespective of these differences, findings from this and our previous investigation demonstrate its successful ability to sustain energy expenditure levels near basal rates, independent of what diet program was being followed (Figure 5).

As we reported previously and as expected, within-group improvements in relative aerobic capacity and relative maximal strength occurred in those groups who followed a diet and exercised [3,21]. While absolute aerobic capacity (L/min) and strength (1RM) did increase (data not shown) in the no diet + exercise and HED groups, the lack of body mass change precluded statistical significance when represented relative to the body mass changes that occurred (or didn't occur) in these groups. In addition to the changes in fitness status, serum values for total cholesterol, LDL cholesterol and triglycerides decreased non-significantly from the beginning to the end of the protocol (Table 4). Overall the magnitude of changes are less than what has been previously reported [5,6], but inherent differences in the study designs (presence of exercise program, length of study, protein type and quality, dietary compliance, etc.) in the investigative approaches may explain some of these differences. Overall it remains that regular participation in a diet and exercise and program can help maintain serum and/or promote improvements in serum based measures of cholesterol metabolism and cardiovascular health.

Great interest has developed into understanding the impact of leptin regulation in conjunction with weight loss, energy expenditure and insulin sensitivity [17,42,43]. It has been well documented that circulating leptin decreases in response to a decreases in energy availability [42], however, the influence of exercise and alterations in the macronutrient ratio is still under-researched. As expected, circulating leptin in the present study decreased in all groups who restricted caloric intake, but no differences relative to macronutrient ratio were determined. Volek et al. [17] reported significant decreases in leptin after an 8-week weight loss program; a finding similar to other studies [3] and the present study. Using a combined diet and exercise approach, Sartorio et al. [42] reported acute, significant reduction in leptin which closely mimicked body mass changes; again a finding supported by the present study and our previous reports [3]. Briefly, previous studies have suggested that diets which contain a higher proportion of dietary protein may promote homeostasis of glucose and insulin [7]. In support of these findings, fasting insulin levels in the VLCHP group were the only group to experience significant reductions from baseline (Table 4) after following their prescribed diet and exercise program after 14 weeks. This response also led to a subsequent significant decrease in HOMA-IR, a homeostatic model of insulin resistance. No significant changes in serum levels of ketones and cortisol were found (Table 4).

The overall safety of higher protein diets has been questioned in the literature. Findings from the current study support findings from our previous investigation using a similar investigative approach that no significant changes occurred in any diet or exercise groups for various markers of kidney and liver function as well as various markers of protein breakdown, which provide additional support for higher protein diets in conjunction with a regular exercise program (data not shown). Nonetheless, it remains that this study and multiple previously published investigations provide evidence that a higher relative protein intake do not invoke negative alterations in any of these serum variables in sedentary, obese, but otherwise healthy populations [1,17,39,44].

## Conclusions

The dietary approaches and exercise program employed through this investigation successfully stimulated significant decreases in waist circumference, body mass, and fat mass, which resulted in an overall improvement in body composition. While other dieting programs have resulted in a decrease in overall energy expenditure in response to acute reductions in energy intake (1,200 kcals•d^-1^), results of the present study indicate that while resting energy expenditure decreased following the first week of dieting, the exercise and dietary approach employed was effective in allowing resting energy expenditure to return toward baseline during the subsequent nine weeks of dieting and during the maintenance phase. Serum levels of cholesterol non-significantly improved while fasting insulin values were significantly improved in those individuals who replaced dietary carbohydrate with dietary protein to the greater extent (VLCHP) and serum leptin levels were significantly improved in all groups that restricted energy intake and followed the exercise program. As a result, when sedentary, obese women followed a restricted energy intake diet, independent of macronutrient distribution, along with a weekly exercise program over a period of 14 weeks, significant reductions in waist circumference, body mass, and improvements in body composition resulted along with general improvements in fitness status and serum markers of energy regulation without negative changes in various markers of clinical safety. While findings from the present study and others help to elucidate the changes seen in healthy but obese women, minimal data exists for pre-diabetic and type 2 diabetic populations. In this regard, future studies should consider investigating varying combinations of diets which restrict energy intake and varying proportions of the macronutrients along with a regular exercise program over the course of several months to determine the efficacy of these interventions in metabolically challenged and other clinically significant populations.

## Competing interests

The authors declare that they have no competing interests.

## Authors' contributions

**CMK, JW, DF, ART, BIC, CDW, TH, MDR, PL, MG, BM: **Assisted with all aspects of data collection and presentation of the study for entire duration of study.**CMK: **Assisted with study development. Prepared final manuscript. **JW, DF: **Served as study coordinators. **ART: **Lead dietician and developed all dietary booklets, dietary familiarizations and was primary contact for all participants on all diet-related questions.**LT: **Coordinated all blood-based analyses. **MG: **Served as research nurse and clinical contact for all study participants completing all medical screening and clearance. **CJR: **Served as laboratory coordinator managing daily operations for all investigations. **RBK: **Principal investigator of the study and was primarily responsible for study development and concept and oversaw all aspects of grant management, personnel considerations and study conductions. **All authors: **Proofed and approved final manuscript.
